# The therapeutic potential of *Ziziphus jujuba* in colorectal cancer: An *in-vitro* study

**DOI:** 10.22038/ajp.2025.26108

**Published:** 2026

**Authors:** Ghazaleh Pourali, Mehrdad Moetamani-Ahmadi, Maryam Alaei, Hamid Fiuji, Alireza Fathi, Mina Maftooh, Majid Khazaei, Gordon A Ferns, Seyed Mahdi Hassanian, Amir Avan

**Affiliations:** 1 *Metabolic Syndrome Research Center, Mashhad University of Medical Sciences, Mashhad, Iran*; 2 *Medical Genetics Research Center, Mashhad University of Medical Sciences, Mashhad, Iran*; 3 *Department of Medical Oncology, Cancer Center Amsterdam, Amsterdam U.M.C., VU, University Medical Center (VUMC), Amsterdam, The Netherlands.*; 4 *Simon Fraser University, Burnaby, British Columbia, Canada.*; 5 *Brighton Sussex Medical School, Division of Medical Education, Falmer, Brighton, Sussex BN1 9PH, UK*

**Keywords:** Colorectal cancer, Ziziphus jujube, Treatments, Anti-inflammatory effects

## Abstract

**Objective:**

Colorectal cancer (CRC) is among the most common causes of death. Thus, identification of innovative therapeutic agents to increase the efficacy of current treatments is needed. The activity of *Ziziphus jujuba* Mill. (Z. *jujuba*) has been reported in several malignancies. Here, we examined the therapeutic potential of Z. *jujuba* in CRC *in vitro*.

**Materials and Methods:**

The anti-proliferative activity of extracted Z. *jujuba* (extracted via hydroalcoholic extraction method) was explored by MTT at 72 hr in CT-26 and SW-480 cells, while wound-healing assays was used to assess its anti-migratory effects t IC50 values of ~500 µg. The anti-tumor activity was investigated using a three-dimensional cell culture model, followed by RT-PCR after 72 hr, and LC/MSMS. Docking analysis was also performed to investigate the interactions between key Z. *jujube *compounds with target proteins.

**Results:**

Z. *jujuba* suppressed cell proliferation and migration by the perturbation of CyclinD1/Survivin and E-cadherin/matrix metalloproteinase 9, respectively. Moreover, treatment of CRC cells with Z. *jujuba* was associated with a reduction in the expression of tumor necrosis factor-alpha and interleukin-6. Moreover, Z. *jujuba* increased pro-apoptotic factors caspas3 and caspase9.

**Conclusion:**

The results demonstrated the therapeutic potential of Z. *jujuba *in CRC through anti-proliferative, and anti-inflammatory properties, indicating its potential value in the treatment of CRC.

## Introduction

The rate of prevalence and death by colorectal cancer (CRC) is increasing (Sung et al. 2021). Chemotherapy remains the standard treatment for CRC patients despite considerable breakthroughs in therapeutic strategies (Goldberg 2005; Mármol et al. 2017). Systemic toxicity and resistance to therapy restrict the effectiveness of current conventional chemotherapy regimens (Longley et al. 2006; Xie et al. 2020). As a result, researchers have tried developing novel substances as supplemental or alternative medicines.

For many diseases, medicinal plants have been utilized as safe, affordable, and accessible therapeutic agents for centuries. Recently, the tendency has returned to using herbal medicines with, or instead of synthetic medications (Khan and Ahmad 2019). The use of novel drugs, including medicinal plants with anticancer benefits, might lead to an improvement in cancer therapy, fewer adverse reactions, and reduced toxicity (Mans et al. 2000). *Ziziphus jujuba* Mill. (Z. *jujuba*), also known as red date or Chinese date, belongs to the Rhamnaceae family, a small shrub that grows in the tropics and subtropical regions (Shahrajabian et al. 2019). It is mostly found in South and East Asia, along with Australia and Europe. Two species are *Ziziphus mauritiana* Lam. and *Ziziphu jujuba* Mill. (Ji et al. 2017; Tripathi 2014). The dark red fruit *Z. jujuba* contains amino acids, polysaccharides, cerebrosides (Kumar et al. 2009). Numerous pharmacological characteristics, such as anti-inflammatory/bacterial/allergy/oxidant/cancer effects have been described (Ji et al. 2019a; Kim et al. 2020; Liang et al. 2020; Resim et al. 2020). There has recently been increasing interest in the potential therapeutic role of Z. *jujuba* in cancer treatment (Aafi et al. 2022). There is some evidence suggesting that this plant may have anti-cancer on different of cancers, including breast (Hoshyar et al. 2015b), melanoma (Hung et al. 2012), and liver cancer cells (Huang et al. 2007). Substances in *Z. jujuba* may be able to stop breast cancer cells proliferation (Plastina et al. 2012), and prevent CRC mice colon shortening, decrease their mortality, and reduce pro-inflammatory cytokines in colon cancer (Ji et al. 2019b). Additionally, Z. *jujuba* was able to induce apoptosis in leukemia cells (Vahedi et al. 2008) and suppress melanoma cells (Hung et al. 2012). However, potential impacts on CRC cells have not been well investigated. In one study, the jujuboside B led to apoptosis of CRC cells by preventing them from proliferating (Xu et al. 2014a). Another study found that betulinic acid, a compound found in jujube, had anti-cancer effects on HCT116 and SW480 cells (Yurasakpong et al. 2021). Therefore, we aimed to explore the therapeutic role of Z. *jujuba* in an *in vitro* model of CRC. We hypothesized that Z. *jujuba* may have anticancer effects on CRC cells by inducing apoptosis, reducing migration, and inhibiting cell proliferation.

## Materials and Methods

### Drugs and chemicals

Z. *jujuba* was purchased from a herbal grocery in Khorasan-Razavi province and identified by botanists in the herbarium of the Pharmacy Faculty, Ferdowsi University of Mashhad (E-1593 FUMH). The process of extracting jujube involved completely drying it in the shade. Then, 100 g of jujube fruit powder was mixed with 1000 ml of distilled water containing 20% ethanol. For 72 hr, the mixture was kept at 33 ^o^C in an incubator. The extract was then run through a filter paper to get rid of any remaining tiny particles. After the solvent was evaporated using a rotary machine, the resultant solution was put in an incubator set at 37 ^o^C. The final product obtained contained 30 to 40 g of dry extract. We then used this power to prepare different concentrations of Z. *jujube* in Dimethyl sulfoxide (DMSO) solvent (Goli-malekabadi et al. 2014; Wang et al. 2010). 

### Cell culture

CT-26 and SW-480 were grown in either Roswell Park Memorial Institute Medium 1640 or Dulbecco's Modified Eagle Medium (DMEM) media (Gibco, Waltham, MA, USA), plus 10% Fetal bovine serum (FBS) (Euroclone, Milan, Italy) and kept at 37°C-5% CO_2_.

### Growth inhibition studies

After 72 hr of treatment, the impact of *Z.* *jujuba* extract on cell proliferation was measured by MTT assay (Hashemzehi et al. 2021). *Z. jujuba* was applied to cell lines at increasing dosages (200, 400, 600, 800, and 1000 µg). Lastly, an ELISA reader (Epoch, BioTek, Winooski, Vermont, USA) was utilized to measure at 570 nm and calculated based on normalization to untreated cells.

### Spheroid analysis

The spheroid of each cell types was formed by seeding 10^5^ cells/ml in 96-well plates precoated with 1% agarose. The spheroids were treated with Z. *jujuba* extract at IC50 value and were assessed using an inverted phase-contrast microscope. Spheroids parameters i.e. diameter, and volume were captured every day and calculated with ImageJ (Friedrich et al. 2009; Hashemzehi et al. 2021).

### Cell migration assay

The antiproliferative activity of *Z. jujuba* on CRC cells was assessed by the scratch-wound assay (Giovannetti et al. 2014). Images were taken at the beginning (Day 0) and up to 72 hr following the treatment of the cells.

### qRT-PCR

Total RNA was extracted (Parstous, Tehran, Iran) after treatment with *Z. jujuba* at IC50 value for 72 hr. Using a cDNA synthesis kit (Parstous, Tehran, Iran), the RNAs were reverse transcribed. Using an ABI-PRISM StepOneTM equipment, qRT-PCR was performed for survivin, Cyclin D1, E-Cadherin, MMP9, caspas3, and caspase9 (Macrogene Co.) and SYBR green master mix (Parstous Co., Tehran, Iran). As an internal control, the housekeeping gene, GAPDH, was employed.

### Molecular docking

We then investigated the interaction of four of the most important compounds in *Z. jujube* with some proteins, involved in cell apoptosis or cell proliferation. Therefore, based on the study of Gao et al. (2013), four compounds namely, cinnamic acid and 4-hydroxybenzoic acid as two phenolic acids, as well as quercetin , and zizyberenalic acid as two flavonoids were assayed for interaction with caspase 3, caspase 9, cyclin D1, E-cadherin, IL-6, MMP3, MMP9, survivin, and TNF-beta. In this method, the bioactive compounds were as ligands and the above macromolecules were as receptors. ChemDraw Ultra software was applied for design of ligands and their geometry were optimized by applying HyperChem program version 8. The crystal structures of with caspase 3 (PDB ID: 2XYG), caspase 9 (PDB ID: 1NW9), cyclin D1 (PDB ID: 2W96), E-cadherin (PDB ID: 2O72), IL-6 (PDB ID: 1ALU), MMP3 (PDB ID: 1D8F), MMP9 (PDB ID: 6ESM), survivin (PDB ID: 1XOX), and TNF-beta (PDB ID: 1TNR) were extracted from Protein Data Bank. To dock ligands with target proteins, the Molecular Operating Environment (MOE) 2019 docking tool was utilized. This is the reason that only the polar hydrogen atoms were added to the molecules and all of the water molecules were eliminated. Using the three-point triangle matching technique, ligands were taken to be flexible molecules in order to determine the optimally optimized interaction. This study made use of the protein's global structure. Ultimately, the lowest docking energy result was considered the option for the ligand-protein characteristics, and Discovery Studio Visualizer 2.5.5 programs were used. 

### Statistical analysis

The data is presented as mean±SEM. The analyses were conducted using GraphPad Prism software. A p-value of less than 0.05 was considered to be statistically significant.

## Results

### Z. jujuba reduces cell growth of CRC cells

**Figure 1:**
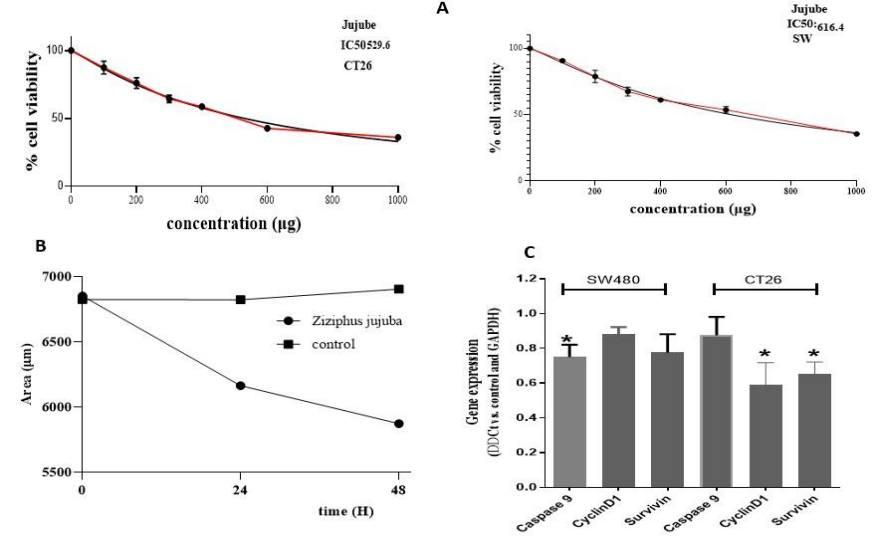
*Ziziphus jujuba* suppresses the cell proliferation. (A) Growing inhibitory properties of *Ziziphus jujuba* on CT26 and SW480 cells after 24 hr, compared to the red line as adjusted line, genereated by Prism. (B) Results of CT-26 spheroids cells. Cells were exposed to *Ziziphus jujuba* at 5xIC50 values. (C) Expression of Caspase 3, Caspase 9, CyclinD1, and survivin after treatment as detected by RT-PCR. Columns represent mean values obtained from two independent experiments; bars show SEM. *Significantly different from controls, untreated cells at 24hr.

To assess the antiproliferative impact of Z. *jujuba*, CT26 cells were treated with Z. *jujuba* for 24 hr. Our data showed that Z. *jujuba* inhibited CRC cell proliferation dose-dependently (Figure 1A). Moreover, Z. *jujuba* reduced the volume of spheroids in three-dimensional cell culture (Figure 1B). RT-PCR was used to assess the levels of cyclin D1 and survivin in order to examine *Z. jujuba*'s function in the cell cycle. The treated CT-26 cells showed decreased levels of survivin and cyclin D1, as seen in Figures 1C–D. 

### Z. jujuba inhibits the migratory behavior of CRC

**Figure 2:**
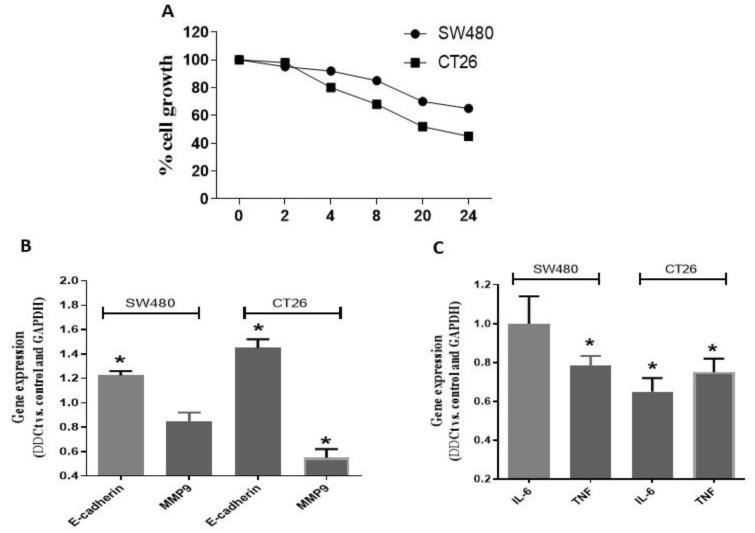
Effects of *Ziziphus jujuba* on cell migration. (A) Results of wound-healing assay in CT-26 cells (insert: representative picture at 0, 24,72 hours). Cells were exposed to* Ziziphus jujuba* at 5xIC50 values. (B) Modulation of E-cadherin, MMP3, and MMP9 mRNA levels after 24 hr exposure as determined by q-RT-PCR. Columns or Points represent mean values obtained from three independent experiments. (C) Expression of IL-6 and TNF-α after treatment with *Ziziphus jujuba.*

To assess Z. *jujuba*'s anti-migratory effects, we performed the scratch test. Results demonstrated that after 24 hr of treatment with Z. *jujuba*, CRC cells' migratory behavior had decreased (Figure 2A). E-Cadherin and MMP9 levels were assessed using quantitative real-time PCR to investigate the underlying processes. It was shown that Z. *jujuba* inhibited cell migration by increasing E-cadherin and decreasing MMP9 (Figure 2B). 

### Z. jujuba modulates apoptosis and inflammatory response in CRC cells

**Figure 3:**
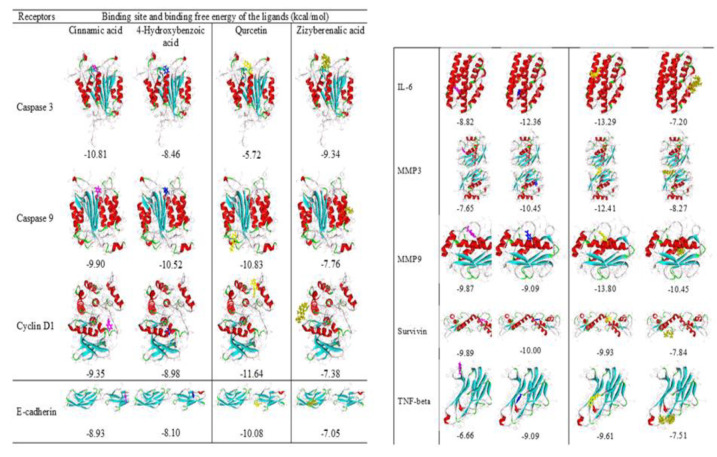
Binding site and binding free energy between ligand and receptor. Each ligand is presented with specific color, as follows cinnamic acid: purple, 4-hydroxybenzoic acid: blue, Quercetin: yellow, and zizyberenalic acid: beige. The binding energy for all complexes is shown in the box.

To examine the potential role of Z. *jujuba* on apoptosis and inflammation, the protein levels of caspas3/9, IL-6, and TNF-α were evaluated. These data showed that the *Z. jujuba* treatment group exhibited elevated caspas3 and caspase9 and downregulated IL-6 and TNF-α versus control group (Figure 3A-B).

### Interactions of Z. jujube with target proteins

**Figure 4:**
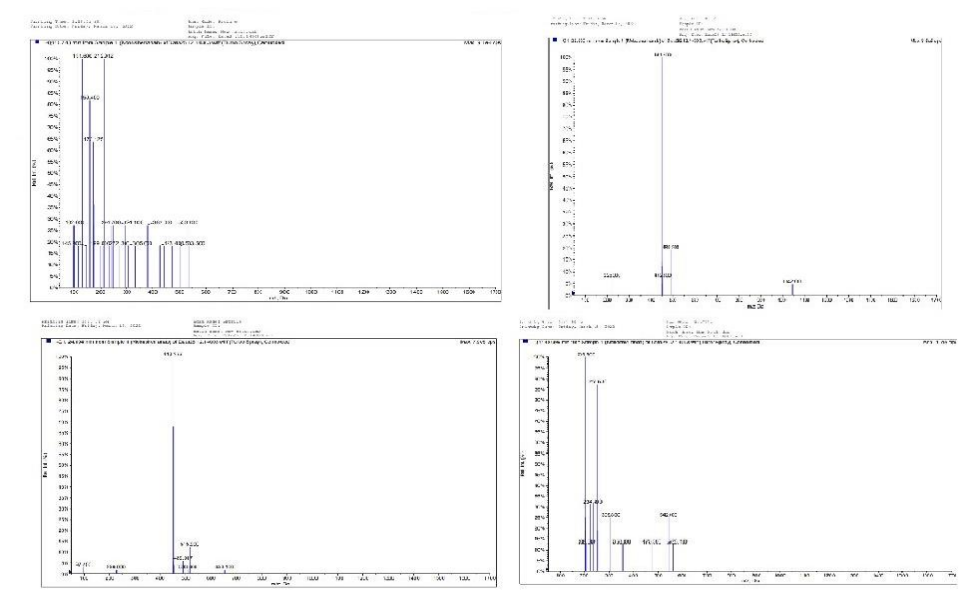
LC-MSMS analysis of* Ziziphus jujuba. *The total ion chromatogram of Z. *jujube* extract as well as the extracted ion chromatograms from the total ion chromatogram and its related mass are shown in Figure 4.

Figure 4 exhibits the results of the bioactive compounds interaction with the receptors. When the binding energy is <-5.0 kcal/mol, the ligand has a good binding affinity to the macromolecules, while levels <-7.0 kcal/mol show a strong binding affinity. The lowest amounts of free energies were related to the interaction of Quercetin with caspase 9 (-10.83 kcal/mol), cyclin D1 (-11.64 kcal/mol), E-cadherin (-10.08 kcal/mol), IL-6 (-13.29 kcal/mol), MMP3 (-12.41 kcal/mol), MMP9 (-13.80 kcal/mol), and TNF-beta (-9.61 kcal/mol). Furthermore, the binding free energy between Quercetin with caspas3 and survivin was -5.72 kcal/mol and 9.93 kcal/mol, respectively. Therefore, based on the docking results and reported as active compound (Khalili-Tanha et al. 2024), it is suggested that quercetin is an active compound with potential ability to bind to important molecules involved in apoptosis or cellular processes. 

## Discussion

Herbal medications are becoming more and more popular among people all over the world due to their low side effects, accessibility, and cost (George 2011);(Kumar et al. 2013). Z. *jujuba* is a popular plant which is widely consumed as a complementary medical agent for its anti-inflammatory and anti-cancer properties all around the world (Shankar et al. 2021). According to recent research, it has strong anti-cancer qualities and can stop the growth of several cancer kinds, including colorectal cancer (CRC) (Ebrahimi et al. 2017). In 2015, Liu et al. examined how dietary *Z. jujube* prevented mice from developing colon cancer associated with colitis. The outcomes demonstrated that this therapy reduced the development of Rectal aberrant crypt foci (ACF) and slowed the transition from hyperplasia to dysplasia (Periasamy et al. 2015). Another study showed that jujuboside B prevented the development of tumors in a xerograph model made up of HCT 116 cells (Xu et al. 2014b). Guo et al. discovered ten triterpenic acids—ceanothic, ziziberanalic acid and ursolic acid, in the dried jujube fruit (Guo et al. 2009). Of all the substances discovered in the dried jujube fruit, a few have cytotoxic effect including betulinic, oleanolic, and ursolic acids (Pisha et al. 1995; Shyu et al. 2010). 

We showed that treatment with Z. *jujuba* exerts its antiproliferative effects via low levels of survivin and cyclin D1. Survivin is considered an apoptosis inhibitor (Li et al. 2015). Survivin is overexpressed in different cancers, including CRC (Hernandez et al. 2011). Another protein that controls the cell cycle's passage from the G1 to the S phase is called cyclin D1, which is frequently overexpressed in cancer cells (Diehl 2002). Cyclin D1 gene is known as a marker of prognosis in CRC (Bahnassy et al. 2004). Consistent with our results, research has demonstrated that administering *Z. jujuba* extract can suppress cyclin D1 expression, resulting in a reduction in proliferation and a rise in cell death in OV-2008 cells (Hoshyar et al. 2015a).

E-cadherin and MMP9 are two important molecules that play a role in the migratory process of CRC (Heslin et al. 2001; Tsanou et al. 2008). E-cadherin is a cell adhesion molecule that helps to maintain the integrity of epithelial tissues by promoting cell-cell adhesion (Van Roy and Berx 2008). In CRC, tumor development and metastasis are correlated with the lack of E-cadherin expression (Yun et al. 2014). On the other hand, MMP9 is a matrix metalloproteinase that degrades extracellular matrix components and promotes tumor invasion and metastasis (Brown and Murray 2015). Some studies have shown that E-cadherin can inhibit MMP9 expression and activity, thereby preventing tumor cell invasion (Nawrocki-Raby et al. 2003). Recent studies have revealed that betulinic acid, a bioactive substance in *Z.* *jujube*, can inhibit colorectal cancer cell lines (Zeng et al. 2019). Betulinic acid induced colorectal cancer cells to undergo apoptosis through increased caspas3, and inhibited the spread of malignant cells via reduced expression levels of MMP (Zeng et al. 2019). In our study, treatment with ***Z.*** *jujuba* extract significantly reduced the migration ability of CT26 cells through E-cadherin and MMP9. This suggests that *Z.* *jujuba* may have anti-metastatic effects by manipulating the levels of these two molecules.

The modification of the apoptotic process is one of the most crucial mechanisms of *Z.* *jujuba*'s anticancer activities (Fulda 2009; Kim et al. 2000). In our analysis of apoptosis-related genes in CRC, we found that using *Z. jujuba* boosts the expression of both casp3/9 (Liu et al. 2015; Olsson and Zhivotovsky 2011). Activation of the executioner caspas3 occurs in the last phases of apoptosis (Galluzzi et al. 2016). In CRC, caspas3 expression is often reduced, which can lead to increased cell growth and resistance to chemotherapy (Zhou et al. 2018). Similarly, caspase9 is an initiator caspase that plays a key role in activating the apoptotic pathway (Galluzzi et al. 2016). In CRC, caspase9 expression is often downregulated, which can contribute to tumor growth and resistance to therapy (Avrutsky and Troy 2021). In line with our findings, jujuboside B, one of the saponins separated from the seeds of *Z.* *jujube*, revealed an antiproliferative effect on HCT 116 via caspas3, FasL, and caspase-8 (Xu et al. 2014b). Similarly, after betulinic acid treatment of HCT116 and SW480 cell lines, pro-apoptotic markers increased (Yurasakpong et al. 2021).

Furthermore, treatment group had considerably lower levels of TNF-α and IL-6 than the control group (Chen et al. 2018). These cytokines can promote the cell growth by stimulating angiogenesis, inhibiting apoptosis (Zhao et al. 2021). A higher risk of developing CRC has been related to chronic inflammation (Long et al. 2017). IL-6 and TNF-α levels are increased in patients with CRC (Komoda et al. 1998; Szlosarek et al. 2006; Xu et al. 2016). Research conducted on murine macrophages in culture revealed that *Z. jujuba* inhibits pro-inflammatory factors such as TNF-α, IL-1β, and IL-6 (Chen et al. 2014). Some studies have suggested that *Z. jujuba* may have anti-cancer effects by reducing inflammation and oxidative stress (Choi et al. 2011; Li et al. 2011; Xue et al. 2009)(Khalili-Tanha et al. 2024).However, there is limited research on whether *Z. jujuba* has any effects on IL-6 and TNF-α levels in CRC. 

In conclusion, a *Z. jujuba* may be a promising therapy for CRC through a variety of possible mechanisms, including lowering tumor cell proliferation, controlling inflammation, reducing migration, and boosting apoptosis. Our data suggested that using the traditional herbal supplement *Z. jujuba* might be a useful therapeutic option for CRC. It is an appealing option for future research as a possible therapeutic treatment for this life threatening illness due to its capacity to control important proteins involved in cell cycle regulation, apoptosis, and inflammation. Since *in-vitro* studies led us to this result, further preclinical research and subsequently randomized clinical trial trials are required to validate the benefits of *Z. jujuba*, discover its detailed anticancer mechanisms, determine optimal dosages and treatment regimens, and evaluate the possible adverse effects.
